# Effect of in-line filtration in newborns: study protocol of the Intravenous Neonatal Central Access Safety (INCAS) randomized controlled trial

**DOI:** 10.1186/s13063-024-08264-w

**Published:** 2024-07-06

**Authors:** Francesco Cresi, Elena Maggiora, Cecilia Capetti, Martina Capitanio, Mattia Ferroglio, Elena Spada, Francesca De Matteis, Sara Cosimi, Fabio Mosca, Alessandra Coscia, INCAS Trial Research Group, Arianna Aceti, Orsola Amato, Gina Ancora, Maria Elisabetta Baldassarre, Giovanni Barone, Cristina Bellan, Gabriella Borgarello, Roberto Bottino, Francesca Campagnoli, Antonella Capasso, Maria Grazia Capretti, Mauro Carpentieri, Roberto Cinelli, Luigi Corvaglia, Simonetta Costa, Carlo Dani, Gabriele D’Amato, Vito D’Andrea, Andrea Dotta, Daniele Farina, Stefania Ferrari, Alessandra Foglianese, Annalisa Fracchiolla, Elena Gallo, Stefano Ghirardello, Nicola Laforgia, Gianluca Lista, Mattia Luciano, Chiara Maddaloni, Gianfranco Maffei, Alessandra Maggio, Luca Maggio, Marzia Maino, Giovanna Mangili, Simona Semeria Mantelli, Raffaele Manzari, Isabella Mondello, Maria Pia Natale, Chiara Peila, Flavia Petrillo, Valentina Pivetti, Federica Pontiggia, Francesco Raimondi, Maria Grazia Romitti, Andrea Sannia, Patrizia Savant Levet, Immacolata Savarese, Ferdinando Spagnuolo, Alessia Varalda, Paolo Ernesto Villani, Gianluca Terrin, Dario Ummarino, Giovanni Vento

**Affiliations:** 1 Neonatal Pathology and Neonatal Intensive Care Unit, University of Turin, Turin, Italy; 2 Città della Salute e della Scienza Di Torino, Turin, Italy; 3 School of Specialization in Pediatrics, University of Turin, Turin, Italy; 4 NICU Fondazione IRCCS Ca’ Granda Ospedale Maggiore Policlinico, Milan, Italy; 5 Department of Clinical Sciences and Community Health, University of Milan, Milan, Italy

**Keywords:** Filtration, Infusion therapy, Newborn, Preterm, Sepsis, CVC-complications, Presepsin, Randomized controlled trial

## Abstract

**Background:**

Particulate contamination due to infusion therapy (administration of parenteral nutrition and medications) carries a potential health risk for infants in neonatal intensive care units (NICUs). This particulate consists of metals, drug crystals, glass fragments, or cotton fibers and can be generated by drug packaging, incomplete reconstitution, and chemical incompatibilities. In-line filters have been shown to remove micro-organisms, endotoxin, air, and particles in critically ill adults and older infants, but its benefits in newborn remain to be demonstrated. Moreover, 50% of inflammatory episodes in the setting of NICUs are blood culture-negative. These episodes could be partly related to the presence of particles in the infusion lines.

**Methods:**

A multicenter randomized single-blind controlled trial was designed. All infants admitted to NICUs for which prolonged infusion therapy is expected will be enrolled in the study and randomized to the Filter or Control arm. All patients will be monitored until discharge, and data will be analyzed according to a “full analysis set.” The primary outcome is the frequency of patients with at least one sepsis-like event, defined by any association of suspected sepsis symptoms with a level of c-reactive protein (CRP) > 5 mg/L in a negative-culture contest. The frequency of sepsis, phlebitis, luminal obstruction, and the duration of mechanical ventilation and of catheter days will be evaluated as secondary outcomes. The sample size was calculated at 368 patients per arm.

**Discussion:**

This is the first multicenter randomized control trial that compares in-line filtration of parenteral nutrition and other intravenous drugs to infusion without filters. Sepsis-like events are commonly diagnosed in clinical practice and are more frequent than sepsis in a positive culture contest. The risk of these episodes in the target population is estimated at 30–35%, but this data is not confirmed in the literature. If the use of in-line filters results in a significant decrease in sepsis-like events and/or in any other complications, the use of in-line filters in all intravenous administration systems may be recommended in NICUs.

**Trial registration:**

ClinicalTrials.gov, NCT05537389, registered on 12 September 2022 (https://classic.clinicaltrials.gov/ct2/show/results/NCT05537389?view=results).

**Supplementary Information:**

The online version contains supplementary material available at 10.1186/s13063-024-08264-w.

## Introduction

### Background and rationale

Intravenous infusion, essential for most of preterm babies, carries the risk for different types of complications such as phlebitis, sepsis, and adverse reactions against materials and fluids introduced into the circulation.

Various studies have demonstrated the contamination of infusion solutions with glass particles from opening glass ampoules, particles from rubber stoppers, or conglomerates of the parenteral nutrition components, including precipitates with inorganic sources containing calcium and phosphorous [1–6]. In an intensive care setting, the particle burden may rise up to one million infused particles per day, increasing with the complexity and quantity of the administered infusions [5].

Information regarding the consequences of particulate infusion derive from animal studies and autopsy reports. Infused particles can block small diameter blood vessels, activate platelets, neutrophils, and endothelial cells and modulate immune response. Heavy particle loads have also been associated with acute respiratory distress syndrome and systemic inflammatory response syndrome [1, 7].

In-line filtration has been proposed as a key practice aimed at enhancing patient safety during parenteral nutrition (PN) administration and/or intravenous drug infusion. Over the years, the focus has shifted away from using filters as a defense against microbial contamination, moving instead toward their ability to remove ever-present particulate matter [1, 4]. Different types of in-line filters with different pore diameters are commercially available. Filters with 0.2 micron are used for aqueous solutions and are expected to stop the vast majority of particles, including bacteria and air bubbles; 1.2-micron filters are used for removing lipid aggregates during administration of PN. In addition, endotoxin retention is reportedly achieved by using a positively charged filter membrane [1, 6].

Although recommendations for the use of in-line intravenous filters in neonates have been published [1, 8], evidence are still insufficient and data are still lacking [9]. To date, in Italy, PN filtration practice vary widely throughout centers, with the majority of neonatal intensive care units (NICUs) using no filters for infusion line. Lack of compliance with filtering guidelines may be related to questions about the strength of available evidence, poor understanding of the risks posed by particulate contamination, a belief that filters are not effective in screening microbes, concerns about costs, and the incidence of clinical problems such as low flow rates and occlusions that may occur during filter use [1].

Sepsis remains an important cause of morbidity in neonatal intensive care units. Central venous catheters (CVCs) and PN are major risk factors [10–12]. Neonatal sepsis has been variously defined according to different clinical and laboratory criteria which make the study of this common and serious condition very difficult. Blood culture remains as the definitive diagnostic tool, though 50% of inflammatory episodes in the setting of NICU are blood culture-negative (sepsis-like events) [13–15]. The sensitivity of blood culture is suboptimal in neonates because of low colony bacteremia, limited blood volume, and inappropriate sampling, but what actually causes sepsis-like events is still debate [13].

We therefore intend to evaluate the effectiveness of in-line filtration in reducing sepsis-like events and other CVC-related complications in preterm and term infants who need prolonged infusion therapy.

### Objectives

The primary objective for the INCAS trial is to determine the effectiveness of in-line filters in reducing the frequency of patients with at least one sepsis-like event, defined by alteration of the biomarkers of inflammation in a negative-culture contest. Further objectives are to evaluate the efficacy of filters in the reduction of sepsis and other CVC-related complications.

### Trial design

The study has been designed as an open multicenter randomized controlled trial. It is a superiority parallel-arm randomized trial (allocation ratio 1:1). The research project will involve a group of NICUs in Italy and will be coordinated by the NICU of the University of Turin.

## Methods: participants, interventions, and outcomes

### Study setting

Patients will be enrolled in the NICU of the coordinating center (Neonatal Intensive Care Unit, University of Turin, Italy) and satellite centers participating in the study. Eligible infants will be identified by the local clinical care team on admission, and they will be screened against the inclusion/exclusions criteria.

### Eligibility criteria

#### Inclusion criteria

Infants will be considered eligible for inclusion into the trial if prolonged infusion therapy (1 week or more) is expected, with either umbilical vein catheters (UVC) in a central position or percutaneously inserted central venous catheter. Infants in whom a peripheral UVC is placed but for whom infusion therapy via central venous access is planned will be enrolled too.

#### Exclusion criteria

Infants will be excluded from participation in the trial if they have clinical characteristics requiring transfer to units not participating in the study before discontinuation of infusion therapy (neurological or surgical diseases, chromosomal abnormalities, and major malformations).

### Who will take informed consent?

Local investigators will recruit patients within 48–72 h from the beginning of infusion therapy and will gain informed consent in a written form. Informed consent will be signed by both parents, and sufficient time will be allowed for consent. Non-Italian-speaking parents will only be asked for their consent if an adult interpreter is available. Trust interpreter and link worker services will be used to support involvement of participants whose first language is not Italian.

## Interventions

### Explanation for the choice of comparators

The study compares the incidence of sepsis-like events and/or other CVC-related complications in two groups of patients: a group in which filters are being used (Filter) and a group in which are not (Control).

### Intervention description

Infants will be allocated to experimental arm (Filter) or to control arm (Control) as per randomization. In the filter arm, all infusions, with the exception of some solutions (blood products), will be subjected to filtration. The aqueous solutions (parenteral therapy and drugs) will be administered through 0.2-μm positively charged filters (Posidyne NEO96E, PALL Medical) which will be replaced every 96 h; the lipid emulsions will be administered through 1.2-μm filters (Lipipor NLF1E Filters, PALL Medical) which will be replaced every 24 h. In the filter arm, all venous accesses (peripheral vein catheters included) will be filtered until infusion therapy suspension. The characteristics of filters are listed in Table 1.

**Table 1:**
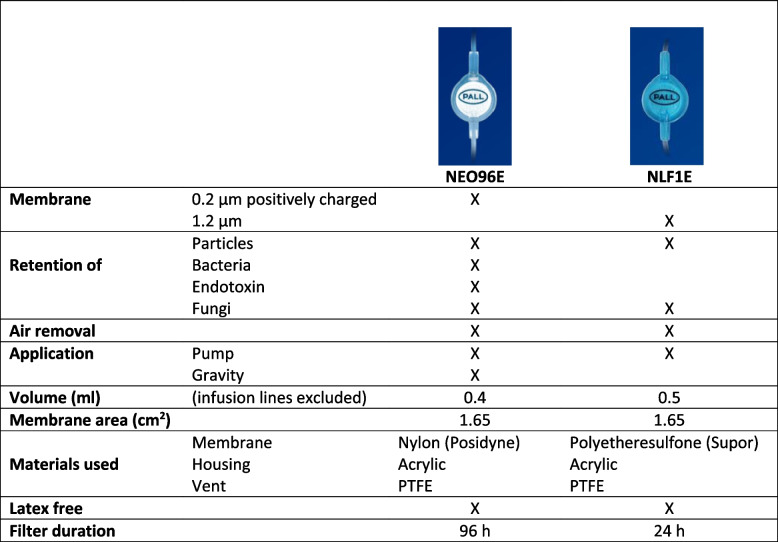
Main characteristics of the filters in the study

*PTFE* Polytetrafluoroethylene

In the control arm, all infusion will be administered through unfiltered accesses.

### Criteria for discontinuing or modifying allocated interventions

1. In case of emergency, life-saving drugs will be administered with bolus modality through the infusion line closer to the patient without the need for filtration.
2. In case of drugs/solutions not supported by filtration (blood products), they will be administered through a dedicated unfiltered access, which will be removed as soon as the drug is no longer needed.
3. Modifying allocated intervention will be tolerated in case of filter unavailability, and in this case, it will be noted in the database.

### Strategies to improve adherence to interventions

Strategies to improve adherence to interventions include staff training performed by Pall Medical Specialists and use of filters for a minimum period of 3 weeks in every center before enrollment begins. The coordinating center will provide flow charts and other instructions, included a document stating drugs compatibility with filtrations.

### Outcomes

The primary outcome is the frequency of patients with at least one sepsis-like event, defined by any association of suspected sepsis symptoms with a level of c-reactive protein (CRP) > 5 mg/L in a negative-culture contest, from the beginning up to 48 h after discontinuation of infusion therapy.

Secondary outcomes are listed below:

- Frequency of patients with at least one inflammatory episode in a positive-culture contest (sepsis events) during infusion therapy.
- Occurrence of phlebitis or local cutaneous inflammation during infusion therapy.
- Occurrence of luminal obstruction and/or extravascular fluid effusion during infusion therapy.
- Duration of mechanical ventilation.
- Number of catheter days.
- Length of stay.
- Neonatal mortality and comorbidity.

### Participant timeline

**Fig. 1:**
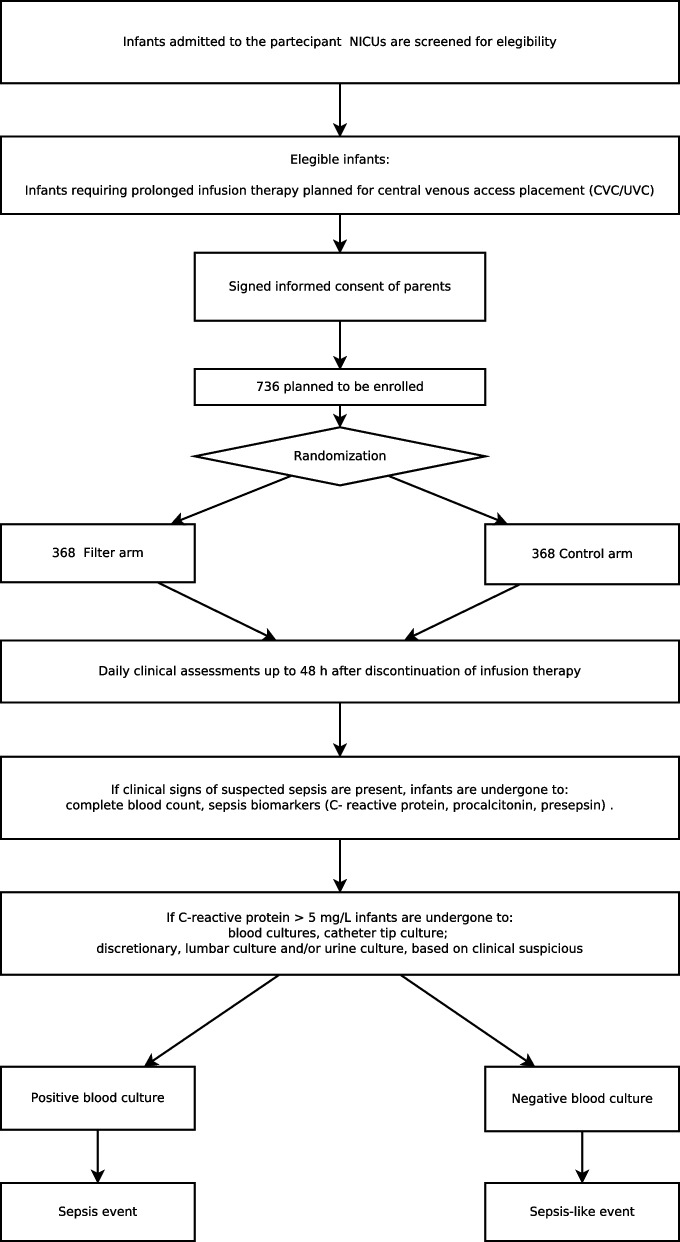
Design of the study

The design of the study is reported in Fig. 1.

### Sample size

The baseline risk of inflammatory states in the target population remains undetermined. However, we hypothesize a range between 30 and 35%. Consequently, a median risk of 32.5% was assumed for the control group. With the application of filters, a plausible 30% risk reduction is anticipated, resulting in an estimated risk of 22.75% in the intervention arm. Utilizing Fisher’s exact test to compare two independent proportions, with an alpha of 0.05, a power of 0.80, and a 1:1 group allocation, the calculated minimum sample size required for significance is 349 infants for each arm, leading to a total of *N* = 698 infants. Accounting for an estimated 5% dropout rate during follow-up, the adjusted minimum sample size becomes *N* = 736 infants.

### Recruitment

Recruitment is anticipated to take 36 months, with 22 sites participating (an average of 1 infant per site, per month over the course of recruitment). All parents of newborn infants for whom prolonged infusion therapy (1 week or more) is expected will be invited to participate in the trial. We planned to complete recruitment before 30/07/2025.

## Assignment of interventions: allocation

### Sequence generation

Eligible patients will be allocated to one of the two arms (Filter or Control) by block randomization. A software has been designed to automatically generate a randomization code and to obtain, in each research unit, a balance between patients of different gestational age (GA) classes (GA ≤ 27w + 6d; GA 28-31w + 6d; GA ≥ 32w). Use of a validated password website will ensure concealment. Infants from multiple births are randomized individually. The website is operational 24 h a day.

### Concealment mechanism

Participants, their families, and clinical and research personnel were unaware of the software generated randomization sequence.

### Implementation

Allocation will be performed by trained members of any local neonatal trial team after obtaining signed consent form and completing the baseline assessment.

## Assignment of interventions: blinding

### Who will be blinded

This will be an unmasked trial; only the statistician will be blinded.

## Data collection and management

### Plans for assessment and collection of outcomes

Prior to this trial, the infusion procedures must be optimized and standardized for all patients. Each research unit will refer to its own protocols for infection surveillance and prevention, although respecting some minimal standard criteria and indications, common and approved by all research units. In each center, the infusion lines must be the same for both intervention arms and differ only in the presence or absence of the filter.

Data will be collected daily from enrolment up to 48 h after discontinuation of infusion therapy.

Each catheter placed in the patients of the two arms will be recorded and at the time of the removal the cause (obstruction, expiration date, etc.) will be specified.

We will request consent for review of participants’ medical records and for the collection of blood samples to assess inflammation: complete blood count, sepsis biomarkers (C- reactive protein, procalcitonin, presepsin), and cultures (blood cultures and/or catheter tip culture; discretionary, lumbar culture and/or urine culture, based on clinical suspicion).

At discharge, information regarding the main neonatal pathologies will be recorded.

Local principal investigators participated in preparatory meeting in which details on study protocol, how to optimize infusion lines, filtering strategies, and data collection were accurately discussed. The full protocol will be available on the INCAS password-protected platform, to which research units will have access. To resolve difficulties, it will be possible to contact the INCAS Trial Coordinating Unit (incas@cresi.org).

### Plans to promote participant retention and complete follow-up

All patients will be followed from recruiting to discharge from the NICU. No follow-up after discharge is planned.

### Data management

All data to be collected will be obtained from the clinical records. Data will be recorded on a common database available on the INCAS website and specifically designed for this study. Access to the database will be password protected, and data will be recorded by trained operators for each center. Participants will be identified by trial number only. The software provides input checks to reduce the possibility of input errors.

### Confidentiality

Data security and anonymity is ensured by the use of access passwords and special encryption procedures.

## Statistical methods

### Statistical methods for primary and secondary outcomes

The trial will be reported using the SPIRIT reporting guidelines.

The analysis will be performed on the “full analysis set” (FAS), i.e., all infants included in the study who did not violate the main requirements of the protocol. Infants whose parents withdrew informed consent, died, or transferred to another center not participating in the study before a time of less than 48 h after the interruption of the infusion therapy, with missing data on the primary outcome, will be excluded from FAS. An additional analysis will be performed on the “per-protocol set” (PPS) including only subjects who did not violate the protocol (infants who did not switch arms and discharged home). Reasons for exclusion from both FAS and PPS will be described in the analysis.

The primary outcome will be assessed using two-tailed Fisher’s exact test (without covariates). Further analysis will be performed using generalized linear models in which the number of catheters/neonate and catheter maintenance time will be considered as adjustment variables. If the sample is unbalanced, gestational age and center will be included as covariates.

Secondary outcomes will be assessed similarly to the primary outcome if possible or with other appropriate generalized linear models.

### Interim analyses

An interim analysis is planned upon reaching the enrollment of half of the patients expected by the sample size calculation.

### Plans to give access to the full protocol, participant-level data, and statistical code

Data will be available with investigator support in deidentified participant data form at the time of the publication of the trial results. Investigators will be able to access data for future ancillary studies and meta-analyses after author approval (INCAS Trial Coordinating Unit).

## Oversight and monitoring

### Composition of the coordinating center and trial steering committee

The coordinating center is composed of a center director (chair) who supervises and guarantees all activities; a principal investigator (PI) who coordinates the activities of all participating centers; some co-investigators involved in consent collection, randomization, data collection, and database entry; an engineer who ensures the security and integrity of the database and a biostatistician who will perform the final analyses. A local PI will be identified in each center, coordinating enrollment and own center data collection.

### Composition of the data monitoring committee, its role and reporting structure

The study protocol has been notified to the Clinical Trial Quality Team of the “Città della Salute e della Scienza di Torino,” who has the authority to stop the trial early if patient safety is compromised and to check procedures accuracy and data integrity.

### Adverse event reporting and harms

This study does not involve the use of drugs or devices capable of causing adverse events. However, the data collection software contains a section for reporting comorbidities and any adverse events.

### Frequency and plans for auditing trial conduct

The INCAS Trial Coordinating Unit is available to help centers every day. Every 3 months, a report will be generated, containing the list of incomplete records, and centers will be notified.

### Plans for communicating important protocol amendments to relevant parties (e.g., trial participants, ethical committees)

Any change to the protocol will be communicated to the ethical committees, and, if approved, it will be presented to the centers on video call and the revisions will be available through the database platform.

### Dissemination plans

The results will be published on international journals and presented at the main national and international congresses.

## Discussion

This is the first multicenter randomized control trial that compares in-line filtration of parenteral nutrition and other intravenous drugs to infusions without filters.

Intravenous in-line filters are currently claimed to be an effective strategy for the removal of bacteria, endotoxins, and particulates associated with intravenous therapy. Several adult studies have shown that intravenous in-line filtration significantly delays the onset of phlebitis, resulting in extended line survival and fewer recannulations, lowers costs, and shortens hospital length of stay [9].

Although recommendations for the use of in-line intravenous filters in neonates have been published, there is no consensus on their use. The American Society of Parenteral and Enteral Nutrition (ASPEN) recommends that healthcare organizations that do not filter parenteral nutrition admixtures or injectable lipid emulsions reevaluate these decisions and consider the small price of filters in comparison to increased morbidity and mortality that may result from not filtering [1]; the most recent edition of the Infusion Nurses Society (INS) clearly affirms that filtration should be adopted when infusing parenteral nutrition solutions [8]; the very recent guidelines from ESPGHAN (European Society for Pediatric Gastroenterology Hepatology and Nutrition) state that in children and neonates, PN solutions should be administered through a terminal filter. Instead, a Cochrane systematic review concluded that there was insufficient evidence for the use of in-line filters in the neonatal population [9].

In neonatal setting, five major studies were published. van Lingen et al. studied the effectiveness of in-line filtration in preventing complications such us bacteremia, phlebitis, extravasation, thrombosis, sepsis, and necrotizing enterocolitis (NEC) in 88 neonates who required an intravenous catheter. They found a decrease in major complications in the filtered group, though no statistical significance was achieved. In addition, incorporating the filter into the intravenous administration system enabled various cost reductions [16]. van den Hoogen et al. results were similar; particularly, they found that nursing time for changing the intravenous sets when using filters was reduced, and a more continuous administration of intravenous medications and parenteral nutrition was guaranteed [17].

Two German studies examined the use of filters in patients admitted to a pediatric intensive care unit (PICU), demonstrating the association between in-line filtration and a reduction in the incidence of SIRS (systemic inflammatory response syndrome) and serious complications [5, 18]. These results though were not confirmed by a recent French study [19]: they compared the profile of pro-inflammatory cytokine levels when using filters or not in 146 very preterm infants and did not find any significant change. The authors conclude that, despite this negative result, several benefits provided by filters should be considered. First, filters can avoid direct infusion of air bubbles or bacteria through central catheters; second, drug incompatibilities and subsequent risk of in-line obstruction can also be prevented.

Sepsis-like events are defined using combined criteria: clinical signs, abnormal laboratory results (RCP ≥ 5 mg/L), but negative blood cultures [13, 20]. They are commonly diagnosed in clinical practice and may be more common than culture-positive sepsis. The risk of sepsis-like events in the target population is estimated to be 30–35%, but this finding is not confirmed in the literature. However, there is growing evidence that these events are a true pathologic condition associated with increased risks of composite of adverse outcomes (brain damage, bronchopulmonary dysplasia), and they may have a noninfectious inflammatory cause, such us particulate.

Our hypothesis is that in-line filtering could reduce sepsis-like events and other CVC-related complications. To these purposes, a multicenter randomized controlled trial was designed. The research project will involve a group of NICUs in Italy and will be coordinated by the NICU of the University of Turin.

If the use of in-line filters demonstrates the expected results, the use of in-line filters in all intravenous administration systems may be recommended in NICU. In addition, the data collected from this study will allow a better definition of the incidence of sepsis-like events and of main CVC-related complications in the Italian context.

## Trial status

The protocol is version 3.0, 9 January 2023. The trial started recruitment on 1 February 2023. The time expected to complete the recruitment is about 36 months (31 July 2025).

### Supplementary Information

Additional file 1. SPIRIT 2013 Checklist: recommended items to address in a clinical trial protocol and related documents.

Additional file 2.

## Data Availability

The dataset generated during the current study is available from the INCAS Trial Coordinating Unit (incas@cresi.org) on reasonable request.

## Abbreviations

ASPEN: American Society of Parenteral and Enteral Nutrition
CRP: C-reactive protein
CVC: Central venous catheter
ESPGHAN: European Society for Pediatric Gastroenterology Hepatology and Nutrition
FAS: Full analysis set
GA: Gestational age
NEC: Necrotizing enterocolitis
NICU: Neonatal intensive care unit
PICU: Pediatric intensive care unit
PN: Parenteral nutrition
PPS: Per-protocol set
SIRS: Systemic inflammatory response syndrome
UVC: Umbilical venous catheter
