# Supplementary material for: Effect of in-line filtration in newborns: study protocol of the Intravenous Neonatal Central Access Safety (INCAS) randomized controlled trial
Source: Trials. 2024 Jul 6;25:459. doi: 10.1186/s13063-024-08264-w (PMC11227126; doi:10.1186/s13063-024-08264-w)
Supplement: Supplementary file 2 — Additional file 2. [file 13063_2024_8264_MOESM2_ESM.docx]

***Spirit figure***


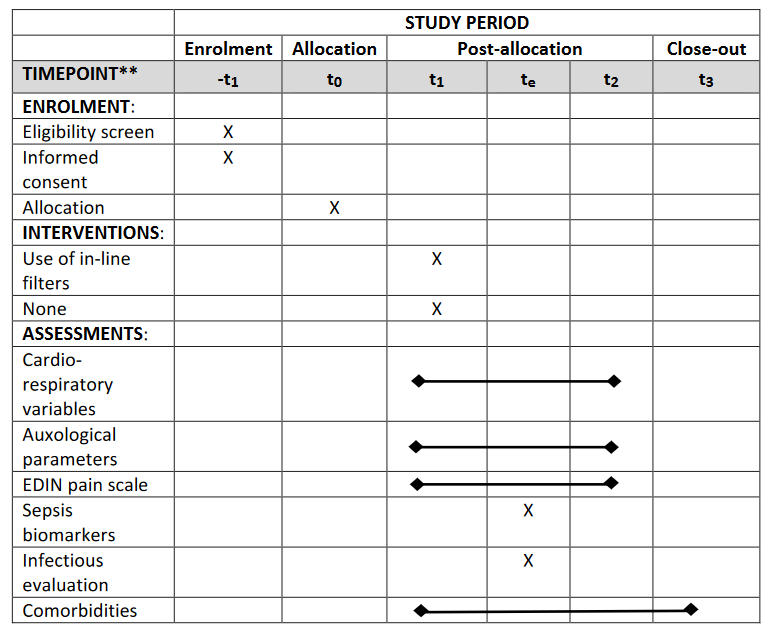


TIMEPOINT**

-t 1: enrollment period, up to 3 days of life

t 0: allocation

t 1: beginning of infusion therapy with or without the use of filters

t e: Sepsis-like event/sepsis

t 2: up to 48 h after discontinuation of infusion therapy

t 3: discharge
